# Mental Health Service Preparedness during a Pandemic: A Delphi Study in Slovenia

**DOI:** 10.2478/sjph-2026-0011

**Published:** 2026-06-01

**Authors:** Špela Selak, Nuša Crnkovič, Janja Horvat, Irena Makivić

**Affiliations:** National Institute of Public Health, Trubarjeva cesta 2, 1000 Ljubljana, Slovenia

**Keywords:** Mental health services, Pandemic preparedness, Delphi consensus, Health system resilience, službe za duševno zdravje, pripravljenost na pandemijo, delfska metoda, odpornost zdravstvenega sistema

## Abstract

**Introduction:**

The COVID-19 pandemic posed significant challenges for mental health, requiring many mental health services to reorganise. The aim of this study was to develop a set of consensus-based recommendations outlining measures and activities for the operation of mental health services during a potential pandemic in Slovenia.

**Methods:**

To develop a preliminary list of measures, relevant literature, existing guidelines, and COVID-19 response practices were reviewed. These measures were then evaluated using a modified Delphi method. Eleven Slovenian mental health experts participated in 4 Delphi rounds to determine which measures should be implemented and at which stage of a potential future pandemic.

**Results:**

Consensus was reached on 47 measures and activities. The majority were classified for implementation before or at the onset of a potential pandemic, with only a small number designated for implementation during the pandemic.

**Conclusions:**

The identified measures and activities are discussed in relation to existing research and international frameworks. They represent consensus-based preparedness guidance for the organisation of mental health services during a pandemic or similar public health emergency and may inform the future development of national guidelines and strategic frameworks.

## Izvleček

**Uvod:**

Pandemija covida-19 je prinesla številne izzive na področju duševnega zdravja, s potrebo po reorganizaciji številnih služb na področju duševnega zdravja. Namen raziskave je bil pripraviti nabor na konsenzu temelječih priporočil, ki opredeljujejo ukrepe in aktivnosti za delovanje služb s področja duševnega zdravja v primeru morebitne prihodnje pandemije v Sloveniji.

**Metode:**

Za pripravo začetnega nabora ukrepov so bili pregledani relevantna literatura, obstoječe smernice in prakse ter odzivi na pandemijo covida-19. Predlagani ukrepi so bili nato ovrednoteni z uporabo modificirane delfske metode. Enajst slovenskih strokovnjakov s področja duševnega zdravja je sodelovalo v štirih krogih delfske študije z namenom opredelitve, katere ukrepe je smiselno uvesti ter v kateri fazi morebitne prihodnje pandemije.

**Rezultati:**

Konsenz je bil dosežen glede 47 ukrepov in aktivnosti. Večina jih je bila opredeljena za izvajanje pred začetkom ali ob začetku morebitne pandemije, le manjši del pa za izvajanje med pandemijo glede na prepoznane potrebe.

**Zaključki:**

Opredeljeni ukrepi in aktivnosti so umeščeni v kontekst obstoječih raziskav in mednarodnih okvirov. Predstavljajo na konsenzu temelječe usmeritve za pripravljenost služb za duševno zdravje v primeru pandemije ali podobne javnozdravstvene krize ter lahko služijo kot izhodišče za razvoj nacionalnih smernic in strateških okvirov.

## 1 INTRODUCTION

From the outset of the COVID-19 pandemic, a mental health crisis was anticipated. As predicted, increases in depression, anxiety, stress, suicidal ideation, and self-harming behaviour were reported globally, including among vulnerable groups, such as adolescents, pregnant and postpartum women, and frontline healthcare workers (1,2,3).

Similar trends were observed in Slovenia, including reduced flourishing and increased languishing mental health (4), as well as higher levels of depression, anxiety, stress, and suicidality among young adults (5) and university students (6).

In response, international organisations urged policymakers to include mental health in COVID-19 response plans (7). The World Health Organization's (WHO) Technical Advisory Group on Mental Health and COVID-19 recommended the continuation of uninterrupted, high-quality mental health care (8). WHO also highlighted the need to integrate mental health services into primary health care during the COVID-19 pandemic (9) as part of broader efforts to strengthen mental health systems and promote, protect, and support mental health at the societal level (10). Access to mental health services was also highlighted by WHO Regional Office for Europe (11), the Organization for Economic Co-operation and Development (OECD) (12), the European Psychiatric Association (13), and the European Public Health Alliance (14). The OECD and the European Psychiatric Association further emphasised integrated and accessible service delivery, including telemedicine, coordination across providers, monitoring of outcomes, and integration of mental health into workplace and educational settings (12, 13).

An international expert group also identified COVID-19 as an opportunity to improve mental health services, highlighting routine outcome measurement, strengthening of primary care, and its integration with secondary care (15).

Crisis response and resilience frameworks provide additional structure for such planning. Mitroff's crisis management model (16), conceptualises crisis response as a sequence of stages, namely detection, preparation, containment, recovery, and learning, and distinguishes between proactive (prevention, preparation, learning) and reactive (coping and recovery) approaches (17). Similarly, WHO defines health system resilience as the capacity to anticipate, prepare for, absorb, adapt to, and recover from crises while maintaining essential services and enabling continuous improvement, emphasising integration, equity, and sustainability (18).

During the pandemic, mental health services worldwide were often unprepared, with disruptions to service delivery, staff shortages, and limited flexibility (2); nearly half of countries reported at least 1 service disruption (10). In Slovenia, the responses included the introduction of psychological support services, reorganisation of care (e.g., telemedicine), and the development of guidelines and support programmes (19, 20). However, significant challenges persisted, including reduced accessibility, staff shortages due to reallocation, insufficient infrastructure, and limited preparedness, with disruptions in preventive and treatment services (19, 21, 22).

Despite existing international guidance and national responses, there remains a lack of structured, consensus-based frameworks that translate recommendations into context-specific operational measures across different pandemic phases. The aim of this study was therefore to develop a set of recommendations for the operation of mental health services during a potential pandemic in Slovenia and beyond.

## 2 METHOD

### 2.1 The Delphi method

This study utilised the Delphi method, an iterative process of anonymous expert consultation using a structured set of questions and controlled feedback to achieve consensus (23). The method is commonly used in contexts characterised by insufficient knowledge, uncertainty, or a lack of evidence, particularly in healthcare (24, 25). A modified online Delphi method, combining qualitative and quantitative approaches, was employed (hereafter referred to as the Delphi study).

### 2.2 Participants

Participants were selected using non-random purposive sampling based on predefined criteria related to professional roles in mental health, including professional background, level of care, mental health service type, and target population. The Delphi panel was designed to ensure a consistent level of expertise while capturing heterogeneity across key dimensions of the Slovenian mental health system.

All invited participants were experienced professionals involved in decision-making or service provision, and all agreed to participate. The final sample was considered sufficient to capture perspectives across levels of care and service types. Regional diversity was considered, while specific locations were concealed to ensure anonymity.

Eleven experts (6 men and 5 women) participated, consistent with recommendations for Delphi panels of 8–12 participants (26). Given the highly specialised nature of the expert field, a smaller sample was considered appropriate to provide reliable and in-depth expert input (27). Participant characteristics are presented in Table 1. The response rate across all rounds was 100%.

**Table 1.** Participants' professional and working background

**Professional background**	**Working field**	**Population they are working with**	**Number of experts**
**Public health expert**	National Institute of Public Health	Children, adolescents and adults	3

**Child and adolescent psychiatrist**	University medical centre	Children and adolescents	1

**Psychiatrist**	Community mental health centre	Adults	1
	Psychiatric hospital	Adults	1
	Psychiatric clinic	Adults	1

**Psychologist**	Health promotion centre	Adults	1

**Clinical psychologist**	Community mental health centre	Children and adolescents	1
	General hospital	Adults	1
	University Rehabilitation Institute of the Republic of Slovenia	Adults	1

### 2.3 Ethical considerations

Participation in the Delphi study was voluntary, and all participants were informed about the purpose and procedures of the study before taking part. Responses were collected anonymously and handled confidentially. Informed consent was implied through participation and completion of all Delphi rounds.

### 2.4 Procedure and data analysis

The proposed activities were developed based on: 1) a previous Slovene Delphi study on mental health needs assessment during the COVID-19 pandemic (28), 2) analyses of established national and international activities during the pandemic (29), 3) an analysis of national directions for mental health service organisation during the pandemic, and 4) research on mental health service functioning in Slovenia during the pandemic (29).

A total of 54 activities were identified across 7 measures or thematic categories: systemic, general, promotion and prevention, treatment, work organisation, education, and telemedicine. Activities were structured according to 2 complementary dimensions: implementation timing (before onset, at onset, and during a pandemic) and thematic categories. These distinguished between system-strengthening measures, which aim to improve baseline system capacity and resilience, and crisis-specific measures, which are activated in response to emergency conditions.

**Figure 1.**
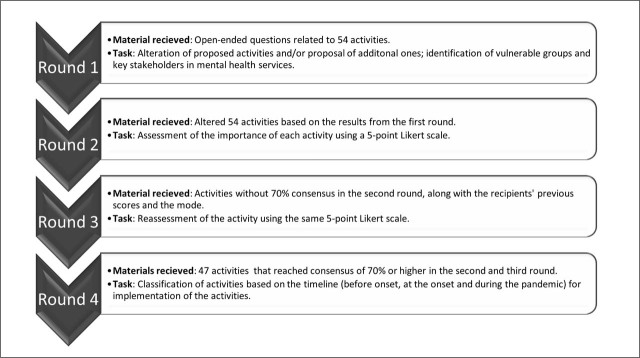
Overview of the Delphi study procedure.

This Delphi study on mental health services during a pandemic was conducted via e-mail and consisted of 4 rounds (Figure 1). In the first round, experts reviewed, added, and modified the proposed activities, and identified vulnerable groups and key stakeholders in the mental health services network to provide contextual mapping of relevant actors and population groups. Responses were analysed using qualitative content analysis, and activities were refined and structured for the second round.

In the second round, participants rated the importance of each activity on a 5-point Likert scale (1 – entirely unimportant, 2 – unimportant, 3 – neither important nor unimportant, 4 – important, 5 – very important). Quantitative data were analysed using descriptive statistics. For each activity, mode and mean scores were calculated, and the level of agreement was expressed as the percentage of participants assigning higher importance ratings. Consensus was defined as at least 70% of panel members rating an item as important (score 4 or 5), corresponding to agreement by at least 8 of 11 experts, a threshold commonly used in Delphi research (30, 31). Sensitivity analysis was conducted using higher thresholds (75% and 80%). Activities meeting consensus threshold were retained.

In the third round, activities that had not reached consensus were reassessed. Participants were provided with their previous ratings and the group modal values from the second round. Consensus was achieved in this round, and activities that did not reach the 70% consensus threshold were excluded. Inter-round stability of ratings was assessed using the interquartile range (IQR) across round two and round three, with smaller values indicating greater convergence of responses.

In the fourth round, activities were classified according to implementation timing using a 3-point scale (1 – should be implemented immediately – before onset; 2 – should be implemented at the onset of a pandemic; 3 – should be implemented during a pandemic according to identified needs). Final categorisation was based on modal responses, appropriate for ordinal data given the prior convergence of ratings.

The final results are presented as a list of activities structured across 3 implementation phases and ranked according to modal expert ratings of importance.

In this study, the term “recommendations” refers to expert-derived proposals for strengthening mental health service preparedness and response, rather than formal national clinical guidelines. Recommendations were conceptually grouped into policy- and system-level measures, organisational procedures, and frontline service activities, and presented according to thematic categories and implementation phases (before onset, at onset, and during a pandemic).

## 3 RESULTS

The results from the first round were analysed using qualitative content analysis. A total of 88 comments on the proposed activities were reviewed and considered, and the proposed activities were revised accordingly. Four activities were modified based on expert input, and 6 additional activities were included and assigned to the relevant measures by the researchers.

The second round yielded 42 activities as important with a consensus of ≥ 70%. Twelve activities that did not meet the consensus threshold were reassessed, with consensus reached for 5. Seven activities were excluded.

Sensitivity analyses applying stricter consensus thresholds of 75%–80% showed that 4 activities would have been excluded after round two and 1 additional activity after round three, compared with the 70% threshold. These findings indicate stability of the retained activity set across alternative consensus thresholds, supporting the use of the 70% consensus criterion.

Although item progression between rounds was determined based on the predefined consensus threshold, dispersion was assessed post hoc through analysis of inter-round stability. Stability of expert ratings across the second and third rounds were assessed using IQR values. From the second to the third Delphi round, IQR values decreased for all items, indicating greater convergence of expert ratings. Of the 47 retained activities, 22 (47%) were classified for implementation before the onset of a potential pandemic and 22 (47%) at its onset. Only 3 activities (6%) were classified for implementation during a pandemic based on identified needs. The final list of activities is presented in Table 2. This structure reflects both the temporal phases of implementation and the distinction between system-strengthening measures (primarily before onset) and crisis-specific measures (at onset and during a pandemic).

**Table 2.** Final consensus-based recommendations for the organisation of mental health services during a pandemic in Slovenia, structured by implementation phase.

**Measure**	**Activity**
**System-strengthening measures – Implementation before the onset of a pandemic**	

**Systemic measures**	Ensure funding for healthcare services (including appropriate costs for labour and materials) and infrastructure (including premises, ICT equipment, vehicles).
	Establish a legal framework for work in the field of mental health (psychotherapy, psychiatry, psychological services).
	Ensure appropriate ventilation of the premises.
	Develop standards for ICT equipment needed for telemedicine.
	Encourage collaboration and coordination among stakeholders, professionals, and service users across all service types and levels; ensure effective design, development, and delivery of services.
	Ensure access to services for all residents of Slovenia across all levels of care.
	Establish preventive services and activities to protect and strengthen public mental health.
	Strengthen services at the primary level of healthcare.
	Strategically plan human resources in the field of healthcare (including backup staff for emergencies).
**General measures**	Establish or adapt cooperation protocols between different mental health services.
	Establish or adapt cross-sectoral stepped care protocols for people with mental health problems.
	Establish or adapt clinical guidelines, clinical and care pathways for treating a range of mental health conditions during a pandemic.
	Establish protocols for access to pharmacotherapy based on the epidemiological situation.
**Promotion and prevention measures**	Implement activities to raise awareness and literacy in the field of mental health.
	Include mental health topics in various professional guidelines.
	Implement destigmatisation initiatives in the field of mental health (with an emphasis on vulnerable groups).
**Education measures**	Provide additional training for mental health professionals in evidence-based interventions to address mental health problems during crisis situations.
	Train non-mental health professionals in psychosocial support, psychological first aid, and the recognition of symptoms of stress and/or trauma.
**Treatment measures**	Expert guidelines for the provision of mental health services during crisis situations have been prepared by relevant professionals.
**Telemedicine measures**	Develop telemedicine as part of the overall system, not as a separate service.
	Train professional staff to deliver services via telemedicine.
	Address potential inequalities in access to telemedicine.

**Crisis-specific measures – Implementation at the onset of a pandemic**	

**Systemic measures**	Ensure sufficient protective equipment for unhindered service delivery in line with professional recommendations.
	Ensure sufficient space for service delivery.
	Encourage, at the level of policy and implementation during a pandemic, the collection, exchange, and use of data among stakeholders, including mental health data.
**General measures**	Identify vulnerable groups and their mental health needs.
	Provide systemic support for identified vulnerable groups and their mental health needs.
	Involve mental health experts in the development of key public messages related to the pandemic.
	Involve mental health experts in pandemic strategy development and measure implementation.
**Work organisation measures**	Introduce staff rotation across work assignments based on stress levels and employee risk factors, while ensuring continuity of multidisciplinary care.
	Pair experienced with less experienced staff (to guarantee intrapersonal support), with the option of regular supervision.
	During escalating situations, ensure continuous and optimal service delivery at all levels of care.
	Minimise, insofar as possible, the redeployment of mental health professionals.
	Provide additional professional support (supervision) for professionals providing psychosocial support.
**Promotion and prevention measures**	Deliver psychoeducational activities for the general population, especially for vulnerable groups.
	Ensure cross-sector collaboration for early detection of mental health issues.
	Provide psychosocial support to the population, especially to vulnerable groups.
	Disseminate professional content for safeguarding and strengthening mental health via various communication channels to different population groups.
**Treatment measures**	Ensure continuity of care for existing patients.
	Ensure continuous psychosocial support for patients, especially for vulnerable groups.
	Follow instructions for preventive (self)protective measures during service delivery.
	Ensure safe, continuous contact of hospitalised patients with their loved ones in accordance with the epidemiological situation and professional recommendations.
	Limit hospitalisations to urgent cases and keep stays short, without compromising the care quality standards.
	Support patients in managing their mental health issues independently.

**Crisis-specific measures – Implementation during a pandemic**	

**Work organisation measures**	If hospitalisations are low, hospital staff should support primary care where emergency cases are treated within 24 hours.
	Organise a hierarchical crisis response based on professional competence profiles and levels of distress.
**Telemedicine measures**	Maintain face-to-face care where possible, with a gradual introduction of hybrid and telemedicine services as the crisis worsens, in line with professional guidelines.

Legend: ICT = Information and communications technology

**Figure 2.**
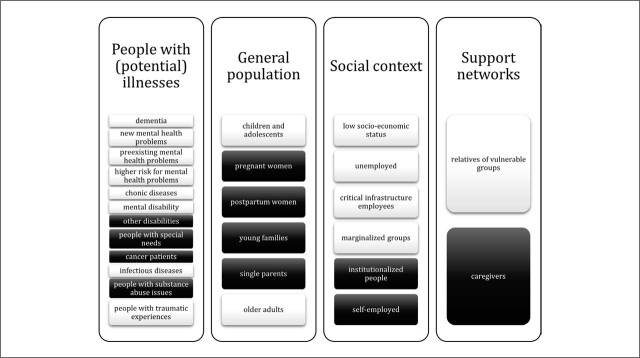
Identified vulnerable groups relevant to the organisation of mental health services during a pandemic in Slovenia.

In addition to rating and refining proposed activities, the Delphi panel identified vulnerable groups (rightsholders) and key stakeholders relevant to the organisation of mental health services during a pandemic. The Delphi process began with 15 vulnerable groups (white) identified in the first round and resulted in 11 additional vulnerable groups (black) in subsequent rounds (Figure 2). These were organised into 4 overarching categories: people with (potential) illnesses, the general population, social context–related groups, and support networks.

**Figure 3.**
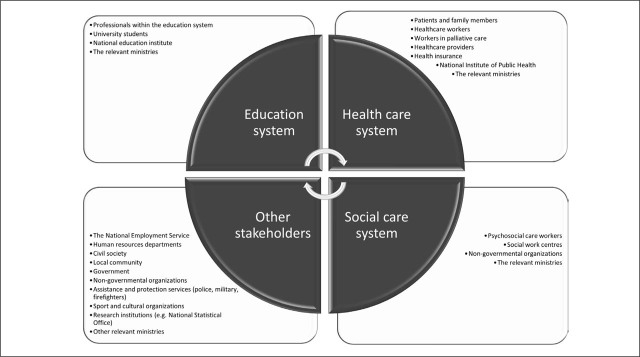
Identified stakeholders relevant to the organisation of mental health services during a pandemic in Slovenia.

Several key stakeholders were also identified through the Delphi process (Figure 3). These were grouped into 4 system-level domains: the healthcare system, social care system, education system, and other cross-sectoral stakeholders. The stakeholder mapping aimed to identify relevant stakeholders involved in the development, coordination, delivery, and implementation of mental health activities before and during a pandemic. This consensus-based identification of stakeholders supports alignment of the proposed recommendations with responsible stakeholders, thereby supporting their feasibility and implementation in practice.

## 4 DISCUSSION

### 4.1 Interpretation of findings in the context of resilience frameworks

The study aimed to develop expert-based recommendations for mental health services in the context of a pandemic by translating established resilience and crisis management frameworks into context-specific operational activities through a structured Delphi process. Although findings reflect the Slovenian context, the approach may inform preparedness planning in comparable healthcare systems by helping to identify system-specific gaps and priorities. Experts identified 47 activities for implementation. The results indicated that almost half of the proposed activities should be implemented before a pandemic and a similar proportion at its onset. This distribution highlights the importance of proactive system strengthening alongside crisis response, consistent with WHO's emphasis on anticipatory resilience-building (18).

Activities identified for immediate implementation were grouped into systemic, general, promotion and prevention, education, treatment, and telemedicine measures. Systemic measures reflect pre-existing structural barriers affecting the efficiency and accessibility of mental health services (28, 32), and align with WHO guidance (7, 8) supporting long-term system strengthening. The shift towards strengthening mental health within primary care is also consistent with international recommendations (9, 15) and supports a more sustainable system. Importantly, these measures reflect structural capacity-building rather than temporary crisis responses. General, education, and telemedicine measures are also consistent with these recommendations (8, 13).

Activities proposed for implementation before pandemic onset align with the initial phases of WHO's resilience framework (18) and Mitroff's crisis management model (16), emphasising prioritisation, preparedness, and early response capacity. Measures such as professional training and the development of clinical guidelines and protocols enhance organisational preparedness and adaptive capacity. These activities may therefore be understood as mechanisms for strengthening adaptive capacity within the mental health system. This is further supported by promotion, prevention, and treatment measures, including continuity of outpatient and community-based care, psychoeducational activities for vulnerable groups, and improved coordination across service levels.

At pandemic onset, systemic and general measures reflect the need for strategic planning and resource mobilisation, consistent with WHO's resilience framework (18) and previous findings of insufficient preparedness (2, 21). Work organisation measures emphasise the protection of healthcare workers, whose increased psychological burden during the COVID-19 pandemic has been well documented (3, 33, 34), highlighting the interdependence between workforce wellbeing and service continuity, a core component of system resilience recognised by WHO (18). Treatment measures focus on maintaining continuity of care, in line with recommendations made by the WHO Technical Advisory Group (8), as service disruptions have been associated with worsening mental health outcomes (2, 35). Participating experts emphasised promotion, prevention, and psychoeducation for the general population, particularly for vulnerable groups identified in this study. In the Slovenia context, existing programmes such as the OMRA programme (36) support baseline health literacy, with additional activities further strengthening awareness and resilience. The emphasis on educational activities for healthcare workers reflects their increased psychosocial burden, particularly among frontline staff. However, they were not explicitly identified as a distinct vulnerable group in the Delphi consensus process, suggesting potential underrepresentation despite strong evidence from COVID-19 literature. These measures aim to contain the impact of the crisis, aligning with crisis management models and supporting system resilience (16, 18), and reinforce preparedness as a continuous, multi-phase process.

The remaining activities focus on accessibility and optimisation of mental health services during a pandemic, including the gradual transition to hybrid and telemedicine delivery of preventive and treatment services, supporting resilience and mitigating the impact of the crisis (16, 18). Among the identified recommendation domains, telemedicine represents a particularly transformative component due to its implications for service delivery and equity. While it supports continuity of care during public health emergencies, its implementation must consider the potential impact of the digital divide (37, 38). Older adults, rural populations, people with low socioeconomic status, and people with severe mental illness may face barriers related to digital literacy, access to devices, internet connectivity, or cognitive and functional limitations (37,38,39). Without targeted support, telepsychiatry and remote services may exacerbate inequalities in access to mental health care (37, 39). These findings highlight that digital innovation should be embedded within equity-oriented health policies rather than implemented as a purely technological solution. Preparedness planning should therefore include strategies to address digital accessibility and literacy before future crises, in line with the WHO Global Strategy on Digital Health (38). This may include digital literacy programmes, alternative access pathways, and monitoring of equity-related indicators in telehealth use.

Although the proposed measures reflect expert consensus and are aligned with established crisis management and health system resilience frameworks, their effectiveness has not been empirically tested. The recommendations should therefore be interpreted as consensus-based guidance that requires further validation through implementation and evaluation.

### 4.2 Operational and system-level implications

In the Slovenian context, surge mental health workforce capacity could be mobilised through temporary redeployment from community mental health centres, health promotion centres, screening programmes, developmental outpatient services, rehabilitation institutes, and other temporarily suspended non-acute services, as well as through coordination with private or contracted providers, non-governmental organisations, and reallocation across different levels of care. Such mobilisation requires predefined protocols, rapid training, and safeguards to maintain continuity of essential services. Stakeholder mapping from the Delphi process clarifies roles and responsibilities across governance levels and supports coordinated implementation of preparedness activities, in line with WHO guidance emphasising clearly defined governance structures, inter-institutional coordination mechanisms, and predefined chains of responsibility at national and regional levels (18). The identification of vulnerable groups and stakeholders should therefore be understood as a contextual mapping exercise rather than as an indicator of differential weighting or causal influence on specific activities.

Although the Delphi process focused on preparedness and response phases, resilience frameworks also emphasise the importance of post-crisis evaluation and learning. These aspects were not explicitly addressed through the expert consensus process and therefore represent an area for further development. Future work should incorporate a post-pandemic evaluation and learning phase, supported by predefined indicators to monitor system performance and inform system-level learning, such as service utilisation, continuity of care, workforce capacity, telemedicine uptake, and equity of access across regions and population groups. Embedding such indicators into routine monitoring could support a shift from reactive crisis response to adaptive learning systems.

The Delphi process primarily engaged mental health professionals; however, the proposed measures reflect a two-pronged approach to pandemic preparedness by addressing both the safety and working conditions of staff and the access to, and the safety of, people receiving care. Measures such as protective equipment, safe working conditions, and telemedicine support infection control while maintaining continuity of care, highlighting their interdependence within resilient health systems.

### 4.3 Methodological considerations and limitations

The study did not include a formal ranking or weighting of activities beyond the consensus threshold, as the aim was to identify core preparedness domains and structure them according to implementation timing rather than to establish a fixed hierarchy of importance. Prioritisation may therefore need to be adapted to national contexts and resource availability. Future research could incorporate structured prioritisation or cost-effectiveness approaches.

Although the Delphi panel was relatively small, it included experts from diverse service settings and levels of care. In the Slovenian context, where the pool of national-level mental health experts is limited, full participation (100% response rate) across all rounds strengthens the robustness of the findings. While the small sample may have limited the range of perspectives captured, the panel size is consistent with Delphi literature (40, 41), and the high level of participant expertise further supports the reliability of the consensus (42).

Several limitations should be acknowledged. The study does not explicitly address post-crisis evaluation and learning, as emphasised in crisis management (16, 17) and resilience frameworks (18). In addition, mental health service users were not involved, and the findings are therefore based solely on expert perspectives, which may introduce bias, as people receiving care could provide additional insights into service organisation and delivery.

Delphi methodology is based on expert consensus, reflecting shared professional judgment rather than proof of effectiveness. The proposed recommendations should therefore be interpreted as informed, expert-based, guidance requiring further validation through real-world implementation and evaluation studies. The proposed recommendations have not yet been empirically implemented or formally evaluated, and their real-world effectiveness and feasibility therefore remain unknown. Future research and practical implementation should incorporate systematic evaluation and feedback mechanisms to support continuous improvement in the organisation of mental health services during public health emergencies.

## 5 CONCLUSIONS

During the COVID-19 pandemic in Slovenia, recommendations for organising mental health services were implemented gradually and without a structured approach, as observed in other European countries (2, 43, 44). This Delphi study identifies pre-existing system barriers in the mental health services and proposes context-specific activities aligned with crisis management and WHO resilience frameworks (16, 18), while tailored to the Slovenian context. It also demonstrates a structured, theory-informed approach to translating international guidance into operational context-specific preparedness activities. Although the findings are specific to the Slovenian context, they may inform preparedness planning in comparable healthcare systems; however, transferability should be interpreted cautiously. The proposed activities should be understood as a foundation for future guideline or strategic framework development rather than as formal clinical or policy guidelines, intended to inform structured preparedness planning processes. Future implementation should include systematic evaluation and integration of service-user perspectives to assess effectiveness, support learning, and refine mental health service responses in future public health emergencies. Future research should incorporate pilot implementation and real-world evaluation of feasibility and impact.
